# Draft Genome Sequence of Bacteriocin-Encoding Enterococcus faecium Strain S6, Isolated from Camel Milk

**DOI:** 10.1128/mra.00888-21

**Published:** 2022-02-17

**Authors:** Abrar Akbar, Rita Rahmeh, Mohamad Kishk, Abdulaziz Alateeqi, Husam Alomirah

**Affiliations:** a Kuwait Institute for Scientific Research, Safat, Kuwait; Loyola University Chicago

## Abstract

Enterococcus faecium strain S6 is a newly identified bacteriocin producer isolated from raw camel milk. The draft genome sequence is composed of 2,617,971 bp, with 2,407 coding genes and a G+C content of 37.99%. The genome sequence analysis provided details into the antimicrobial properties of strain S6.

## ANNOUNCEMENT

Enterococci are Gram-positive lactic acid bacteria that inhabit the gastrointestinal tracts of different hosts (1). Enterococcus faecium strain S6 was isolated from raw camel milk in Kabad, Kuwait (northwest region). The strain exhibited strong antimicrobial activity against three indicator strains, including Listeria monocytogenes, Salmonella enterica, and Escherichia coli (2). Bacteriocins and bacteriocin-producing bacterial strains can be used in the food industry as food preservatives, and they can substitute conventional antibiotics especially in animal food production (3). This report explains the entry of E. faecium, which was isolated from raw camel milk, into the NCBI database genome.

Before isolation, bacteria were grown on MRS medium for 24 h at 37°C under aerobic conditions (4). Genomic DNA was extracted from the cultured bacteria by the GeneElute bacterial genomic DNA kit (Sigma-Aldrich Co. LLC). Barcoded DNA libraries were prepared using the Nextera XT library (Illumina, Inc., San Diego, CA, USA) following the manufacturer’s instructions. The 2 × 250-bp paired-end read sequencing was performed on a HiSeq 2500 instrument (Illumina, Inc.). Read quality was controlled using the FASTQC quality-control tool version 0.11.5. In addition, the sequences were enhanced using the read error correction module BayesHammer in the SPAdes version 3.10 genome assembly tool kit (5), and the high-quality reads were assembled into contigs using SPAdes software. Misassembles and nucleotide disagreement between the Illumina data and the contig sequences were corrected by Pilon (6) version 1.11, which resulted in 37 scaffolds of different sizes (minimum scaffold size, 320 bp; maximum scaffold, 284,269 bp). Genome annotation was performed using the NCBI Prokaryotic Genome Annotation system (PGAP; version 5.2) (7). The genome sequence consists of 2,617,971 bases, with a G+C content of 37.99%. The number of raw reads was 4,437,645, and the assembly *N*_50_ score was 191,551 bp with a 408,49 average coverage. A total of 2,453 coding sequences (CDSs) and 55 structural RNAs were predicted. There were 2,407 CDSs with a known function and 129 with a signal peptide. Several CDSs for the production of bacteriocins, namely, enterocin A, enterocin B, two-component enterocin X (X-alfa and X-beta subunits), and lactococcin, were found using BAGEL4 analysis software (8). Virulence factors, such as surface aggregating-protein, gelatinase, and hyaluronidase, were not detected in the sequence using Virulence Finder software (version 2) (9–11). Furthermore, ResFinder software 3.0 (9, 12, 13) was used to identify acquired resistance genes. As shown in Table 1, the resistance genes are tetracycline, macrolide, lincosamide, and streptogramin B. Unless stated otherwise, default parameters were used for all software tools.

**TABLE 1:** Summary of acquired resistance genes using ResFinder software 3.0

Gene	Identity (%)	Alignment length	Coverage (×)	Contig	Position in contig	Phenotype
*tet*(L)	100	1,377/1,377	100	s6_contig_00016	2478..3854	Tetracycline resistance
*tet*(M)	96.4583	1,920/1,920	100	s6_contig_00016	4048..5967	Tetracycline resistance
*msr*(C)	98.9858	1,479/1,479	100	s6_contig_00007	80264..81742	Macrolide resistance
*lsa*(E)	99.596	1,485/1,485	100	s6_contig_00016	28907..30391	Streptogramin B resistance
*lnu*(B)	99.3781	804/804	100	s6_contig_00016	30445..31248	Lincosamide resistance

This work highlights the potential biotechnological application of this strain for the production of enterocins, which are bacteriocins that can be employed in the food industry as a biopreservative against L. monocytogenes and as an alternative to classical antibiotics.

### Data availability.

This whole-genome sequencing project has been deposited at DDBJ/ENA/GenBank under the accession JAHCYY000000000.1. The version described in this paper is the first version, JAHCYY010000000. Raw sequencing reads are available in NCBI SRA under accession number SRR17078534.
